# *HOXA13 *and *HOXD13 *expression during development of the syndactylous digits in the marsupial *Macropus eugenii*

**DOI:** 10.1186/1471-213X-12-2

**Published:** 2012-01-11

**Authors:** Keng Yih Chew, Hongshi Yu, Andrew J Pask, Geoffrey Shaw, Marilyn B Renfree

**Affiliations:** 1 ARC Centre of Excellence in Kangaroo Genomics, The University of Melbourne, Melbourne, Victoria, 3010, Australia; 2 Department of Zoology, The University of Melbourne, Melbourne, Victoria, 3010, Australia; 3 Department of Molecular and Cellular Biology, The University of Connecticut, Storrs, CT 06269, USA

## Abstract

**Background:**

Kangaroos and wallabies have specialised limbs that allow for their hopping mode of locomotion. The hindlimbs differentiate much later in development but become much larger than the forelimbs. The hindlimb autopod has only four digits, the fourth of which is greatly elongated, while digits two and three are syndactylous. We investigated the expression of two genes, *HOXA13 and HOXD13*, that are crucial for digit patterning in mice during formation of the limbs of the tammar wallaby.

**Results:**

We describe the development of the tammar limbs at key stages before birth. There was marked heterochrony and the hindlimb developed more slowly than the forelimb. Both tammar *HOXA13 *and *HOXD13 *have two exons as in humans, mice and chickens. *HOXA13 *had an early and distal mRNA distribution in the tammar limb bud as in the mouse, but forelimb expression preceded that in the hindlimb. *HOXD13 *mRNA was expressed earlier in the forelimb than the hindlimb and was predominantly detected in the interdigital tissues of the forelimb. In contrast, the hindlimb had a more restricted expression pattern that appeared to be expressed at discrete points at both posterior and anterior margins of the limb bud, and was unlike expression seen in the mouse and the chicken.

**Conclusions:**

This is the first examination of *HOXA *and *HOXD *gene expression in a marsupial. The gene structure and predicted proteins were highly conserved with their eutherian orthologues. Interestingly, despite the morphological differences in hindlimb patterning, there were no modifications to the polyalanine tract of either *HOXA13 *or *HOXD13 *when compared to those of the mouse and bat but there was a marked difference between the tammar and the other mammals in the region of the first polyserine tract of *HOXD13*. There were also altered expression domains for both genes in the developing tammar limbs compared to the chicken and mouse. Together these findings suggest that the timing of *HOX *gene expression may contribute to the heterochrony of the forelimb and hindlimb and that alteration to *HOX *domains may influence phenotypic differences that lead to the development of marsupial syndactylous digits.

## Background

The limbs are highly variable structures between different mammalian and vertebrate species [1] enabling them to adapt and exploit new habitats. The vertebrate limb has served as a key model for understanding the signalling pathways controlling patterning and morphogenesis [2]. Limb patterning genes and pathways have been well described in mice and chickens, but very few other model animals have been examined [1,3,4].

The tammar wallaby, *Macropus eugenii*, like all macropodid marsupials, has large hindlimbs specially adapted for hopping. The digits on the hindlimb are highly modified: digit 1 is never present, but digits 2 and 3 are fused and there is an elongated digit 4 [5]. The tammar delivers an altricial young which climbs to the pouch using its relatively well developed forelimbs, but the hindlimbs are not yet functional and are essentially fetal. After birth, this situation changes and the hindlimb growth and development rapidly overtakes that of the forelimb during early pouch life. Despite this difference in timing, the tammar hindlimb autopod is specified before birth and the early formation of the syndactylous hindlimb digits is already initiated. However, the gene(s) regulating this process are as yet unknown.

Patterning of the vertebrate limb is coordinated by morphogens secreted across three different axes in the early limb bud; proximal distal (PD), dorsal ventral (DV) and anterior posterior (AP) [reviewed by [2]]. These morphogenic gradients dictate the formation of the stylopod (shoulder), zeugopodium (radius and ulna) and autopodium (hand and digits) [reviewed by [6]]. Fibroblast growth factor 8 (FGF8) expressed in the Apical ectodermal ridge (AER) controls a positive feedback signal that dictates PD outgrowth [7]. Bone morphogenetic protein 4 (BMP4) is a key regulator of interdigital webbing and dorsal ventral polarity [8] while sonic hedgehog (SHH) secreted from zone of polarising activity (ZPA) (a signalling centre in the posterior region of the limb bud) acts via a negative feedback loop with BMP4 to determine anterior posterior patterning and digit identity [9],

Interestingly, many of these genes and proteins also play conserved roles in the development of another appendage, the phallus [10,11]. SHH is secreted from the urethral epithelium of the phallus and regulates patterning much like the ZPA in the limb bud [11,12]. BMP4 is expressed in the phallus in the distal region near the urethral epithelium and interacts with SHH [reviewed by [11]]. Although these appendages are phenotypically different, the main signalling pathways remain the same [11] and *Homeobox *(HOX) genes underpin their regulation.

The HOX genes are crucial regulators of embryonic development and the phenotypic differences in the vertebrate body plan [reviewed by [13]] and are responsible for patterning the limb [reviewed by [12,14,15]]. *Homeobox A13 (HOXA13) *and *Homeobox D13 (HOXD13) *are essential for formation of the autopod and digit patterning in the mouse [16]. *HOXA13 *mutants have fused digits and there is no digit 1 (the most anterior digit) [16]. *HOXD13 *mutations result in fusion of digits 3 and 4 and a localised delay in autopod ossification [16]. *HOXA13 *and *HOXD13 *double heterozygous mutants have more severe limb and genital phenotypes compared to either individual gene mutation [16].

Similarly in humans, mutations in *HOXA13 *and *HOXD13 *result in limb and genital malformations such as synpolydactyly, polydactyly and hypospadias [17-19]. Many of these mutations are due to the expansion or reduction of a polyalanine tract in these two genes [20,21], changing the biochemical conformation [22]. Interestingly, the chicken and zebrafish have shorter polyalanine tracts and both possess highly modified appendages [23]. It is still not clear how the *HOX *genes are regulated [14] but human mutations such as Townes-Brocks [24] suggests that the transcription factors SALL1 and SALL3 influence the SHH and HOX pathways [25]. Double null *SALL1/SALL3 *mutants have lost digit 1 and have fused digits 2 and 3, much like the *HOXA13/HOXD13 *double heterozygous mutants [25].

The bat *Carollia perspicillata*, like all chiropterans, has a highly specialised forelimb that evolved to enable flight [4]. There is an expanded and posteriorly-shifted *HOXD13 *expression in the forelimbs compared to expression in the mouse [3,26]. In addition, bats have developed a mechanism of interdigital retention involving the regulation of *BMP4 *and *FGF8 *through *Gremlin *(*GREM1*) [8]. *SHH *has a second wave of expression in the bat forelimb [27] and may reinitiate the loop between FGF and SHH to retain the interdigital webbing of the bat and elongate the forelimb digits [27]. In contrast, the macropodid marsupials have elongated and expanded their hindlimb digits, so a comparison of expression profiles for key limb patterning factors in the tammar may help to define the mechanisms underlying vertebrate limb diversity.

To date there is only limited information about the molecular control of marsupial limb development. The grey short-tailed opossum *Monodelphis domestica *has precocious forelimb development (as is also seen in the tammar) [1,28] that facilitates the crawl from the birth canal to the mammary glands, but the hindlimb development lags slightly behind [29,30]. This heterochronic development of the forelimbs is reflected by the timing of gene expression. Paired-like homeodomain transcription factor 1 (*PITX1*) expression an upstream inducer of T-box 4 (*TBX4*) is expressed late in opossum development relative to the mouse [29]. *TBX4 *and *T-box 5 *(*TBX5*) are markers of hindlimb and forelimb position respectively and in the opossum are expressed relatively early in development, indicating that the opossum forelimb field arises relatively earlier than in the mouse and earlier than the opossum hindlimb [29,31]. However, unlike the tammar, the opossum limbs are not especially modified and have all 5 digits [32].

We therefore focussed on the spatio-temporal changes of two genes, *HOXA13 *and *HOXD13*, known to be essential for digit development in the mouse and chicken, to investigate the possible role of *HOX *genes during digit and limb development in the tammar and to determine whether differential patterning accounts for their unique digit modifications.

## Methods

### Animals

Tammar embryos were collected from a wild population on Kangaroo Island, South Australia. Morphological observations and measurements were made on a collection of 28 embryo and fetus samples. Age was estimated on embryo images and developmental growth curves given in [33-35]. Due to different growth rates and a poor correlation between age and stage in individual animals, there is some variation of limb phenotypes relative to other characters. Age of some specimens have therefore been estimated to within a half day. Fetal samples were collected from day 18 to day 25 of the 26.5 day pregnancy (n = 3 at each stage for *in situ *and PCR except for a single d18 embryo). Tissues for sequencing were obtained from the same individuals as used for the PCR analyses.

Fetal limbs were dissected from the main body trunk and snap frozen for RT-PCR or fixed whole in 4% paraformaldehyde. Three additional animals (2 fetuses at day 23 and 25 of pregnancy and three pouch young at day 3, 120 and 150 post-partum (pp) as well as an adult) were used for cartilage and bone imaging. All sampling techniques and collection of tissues conformed to Australian National Health and Medical Research Council (2004) guidelines and were approved by The University of Melbourne Animal Experimentation & Ethics Committees.

### Extraction and isolation of tammar *HOXA13 *and *HOXD13*

Partial sequences of tammar *HOXA13 *and *HOXD13 *were identified from the NCBI tammar genome trace archives, and the full sequences were obtained by BAC screening and BAC DNA 454 shotgun sequencing (H Yu, Z-P Feng, R O'Neill,Y Hu, A Pask, D Carone, J Lindsay, G Shaw, S Frankenberg, AT Papenfuss and MB Renfree, unpublished data). Primers for RT-PCR and whole mount *in situ *probes were designed to span an intron and exon boundary based on cloned BAC DNA sequence (Table 1). Total RNA was extracted from tammar fetal limbs and cDNA synthesis was performed according to the manufacturer's protocol (Invitrogen, NSW, Australia). To examine *HOXA13 *and *HOXD13 *expression in developing limbs and to make *in situ *templates, RT-PCR was performed using the following conditions: 35 cycles of 30s, 95°C; 30s, 58°C; 60s, 72°C, in a 25 μl reaction with GoTaq Green Master Mix (Promega, Wisconsin, USA). The RNA control was a pooled template before reverse transcribed into cDNA of forelimb or hindlimb.

**Table 1:** Primers used in this study (5' to 3')

Gene	Forward	Reverse	Amplicon size (BP)
HOXA13 (WISH)	CTACTTCGGCAGCGGTTA	TGTCGTCTTGAGTTTGTTGA	676
*HOXD13 *(WISH)	GTTCATCCTCTTCCTCCTC	AATGGCGTATTCGTTCTC	656
HOXA13 (PCR)	ACCTCTGGAAGTCCACTCTG	CTTGGTGTAAGGCACTCGTT	95
*HOXD13 *(PCR)	CTGGACTCTGGCGAATGG	GGTTTGTGGCTGCGGATA	251
M13puc (sequencing)	CCCAGTCACGACGTTGTAAAACG	AGCGGATAACAATTTCACACAGG	

HOXA13 and HOXD13 protein sequences of human, mouse, opossum, platypus, chicken and zebrafish were retrieved from GenBank (http://www.ncbi.nlm.nih.gov/) (Additional file 1, Table S1). The protein sequences were aligned with MUSCLE program [36]. The phylogenetic tree was constructed by neighbour-joint method with default parameter of program MEGA5 [37]. The second structure of HOXD13 N-terminal was predicated using PHYRE http://www.sbg.bio.ic.ac.uk/~phyre/[38] and the second structure is the consensus structure predicated by pispred, jnet and sspro.

### Whole mount *in situ *hybridisation

Whole mount *in situ *hybridisation was performed as described previously [38]. Embryos were fixed in 4% paraformaldehyde at 4°C overnight, washed and stored in 100% methanol at -20°C until analysis. Limbs were dissected from the fetal bodies and rehydrated through a graded methanol series (10 mins each). Fetuses were washed in PBS with 0.1% triton X-100 (PBTX) (3 × 10 mins) before proteinase K treatment 10 ug/ml (30-60 minutes). Fetal limbs were washed in PBTX for (2 × 5) minutes and fixed with 0.2% glutaraldehyde/4% PFA in PBTX for 20 minutes on a rocker. Embryos are then incubated in pre-hybridisation mix at 65°C overnight before 1 ug of a cRNA probe synthesized with T7 or SP6 RNA polymerase (Promega, Australia) was added and incubated overnight at 65°C. Using saline-sodium citrate (ssc)/0.5% 3-[(3-cholamidopropyl) dimethylamino]-1-propanesulfonate (CHAPS), tissues were washed and blocked for 2-3 hours and incubated overnight in alkaline phosphate-conjugated DIG antibody (Roche Diagnostics Corporation, Indianapolis, IN). The specimens were washed in Tris-buffered saline with triton X-100 (4 × 60 mins) before colour development. All specimens were incubated in nitro-blue tetrazolium and 5-bromo-4-chloro-3'-indolyphosphate (NBT/BCIP) for a minimum of 2 hours. Photographs were taken using an Olympus dp25 camera mounted on an Olympus (ZX-9) dissection microscope.

### Bone and cartilage staining

#### Alcian blue staining (fetal stages)

Alcian blue staining of fetal stages was carried out as described by [39] with the following modifications. Specimens were initially fixed in 4% paraformaldehyde (PFA) overnight and then washed and stored in 70% ethanol before staining. Specimens were then washed in a 1:1 mixture of ethanol and NaOH before staining.

#### Alcian blue and alizarin red staining of postnatal bone

All specimens were initially fixed in 4% PFA overnight and then washed and stored in 70% ethanol before staining. As much skin and fat as possible was carefully removed before placing the eviscerated specimen in alcian blue solution (0.05% Alcian Blue 8 GX (Prositech; Queesland, Australia) in 95% ethanol, 5% acetic acid) overnight. The following day the specimens are washed in 95% ethanol overnight and then incubated in 2% KOH for 1-2 days. Specimens are then placed in Alizarin red solution (0.1% alizarin red in 1% KOH) for 2 days. Specimens are then cleared in 1% KOH, 20% glycerol for at least 2 days or until clear before photography.

## Results

### Tammar limb development

**Figure 1:**
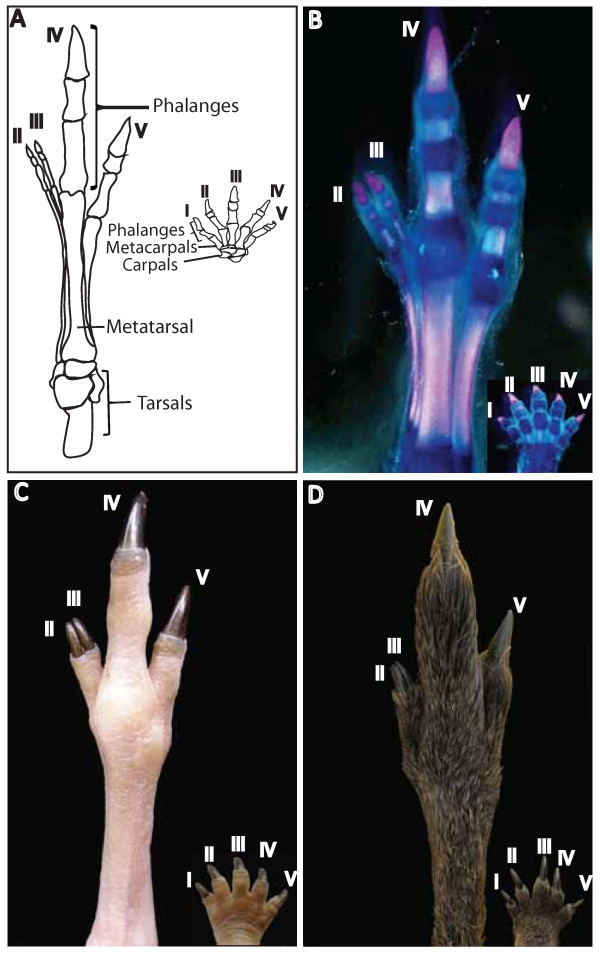
**The morphology of the tammar wallaby autopod (A) Diagram of the macropodid forelimb and hindlimb bones (Adapted from **[53]). (B) A day 150 post-partum (pp) forelimb (inset) and hindlimb stained for bone and cartilage with alcian blue and counter stained with alizarin red. (C) A day 120 pp forelimb (inset) and hindlimb. (D) An adult tammar forelimb (inset) and hindlimb. In the forelimb, all five digits were present. In contrast the most anterior digits of the hindlimb were the syndactylous digits two and three. Digits are numbered with Roman numerals.

**Figure 2:**
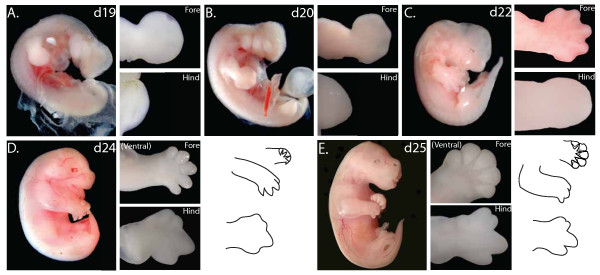
**The development of tammar fetal limbs at selected stages before birth**. (A) day 19, (B) day 20, (C) day 22, (D) day 24 and (E) day 25 (one day before birth). High magnification of the fore- and hindlimb are from samples stored in methanol whilst wholemounts were stored in 70% ethanol. A diagrammatic representation of the fore and hindlimb at day 24 and day 25 is provided showing dorsal and ventral views. All limbs are viewed from the dorsal aspect unless indicated. NB: images not to scale.

The forelimbs of adult tammars are similar to those of the mouse and have five digits making a hand that is well adapted to grasping (Figure 1). However, the tammar hindlimb has only four digits. Tammar digit one is never present at any stage of development whilst digits two and three are fused (syndactyly). The fourth digit is greatly elongated and the fifth digit is reduced. The syndactylous form of the limb is specified between day 23 and day 24 of pregnancy (Figure 2). There are differences in the developmental timing of fore- and hindlimbs.

At day 19 of pregnancy the tammar forelimb formed initially as a bud whilst the hindlimb was a ridge less than half of the forelimb bud size. The forelimb transitioned from a club-shaped bud structure to a paddle between day 19 and day 20 but digital rays were only visible on the dorsal surface towards the end of this stage. The hindlimb developed into a pronounced outgrowth that resembled a bud by day 20-2 of the 26.5 day pregnancy (Figure 2A and 2B).

By day 22, the digital rays of the forelimb were well defined from the mesenchyme with interdigital webbing (Figure 2C). The digits were at an early stage of separation but the marsupial epitrichial claws that are used to assist the climb to the pouch were not yet developed. In contrast, the hindlimb was an asymmetrical bud beginning to form digital condensations.

Epitrichial claws formed at day 24, and the digits had separated, extending forward but were uncurled. The hindlimb had rays formed by digital primordia with a single enlarged digit four that eventually would form the asymmetrical hindlimb. At the most anterior point, digits two and three were distinct but shorter in length than digit 4. This was the first sign of the developing syndactylous digits (Figure 2D).

**Figure 3:**
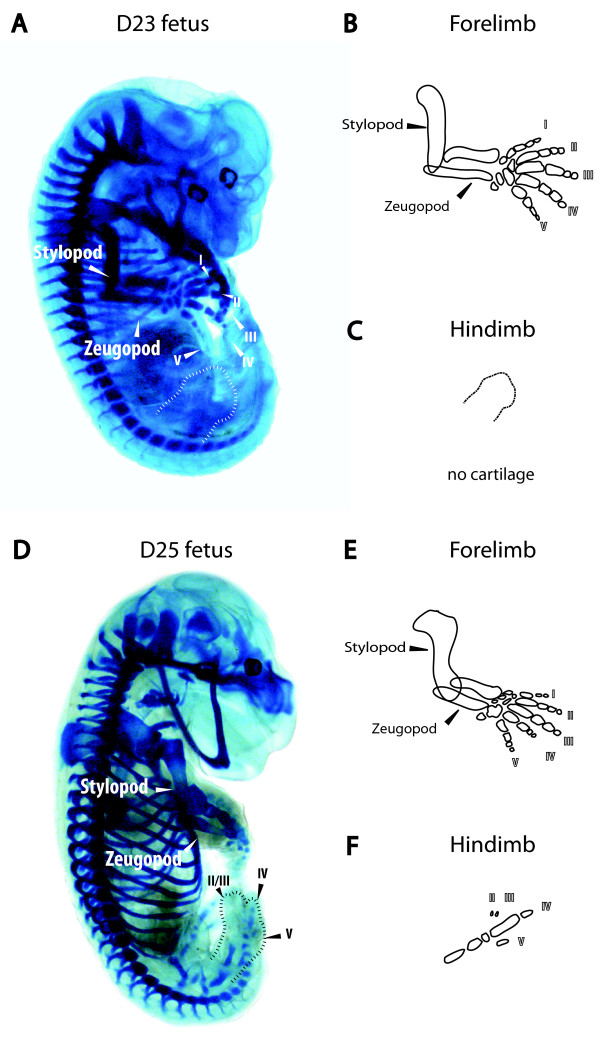
**The development of bone and cartilage in the tammar limb**. Alcian blue staining of (A) day 23 fetus and (D) day 25 fetus. A diagrammatic representation of alcian blue stained cartilage elements in a day 23 fetus (B) forelimb, (C) hindlimb and day 25 fetus (E) forelimb and (F) hindlimb. Staining show the early skeletal element deposited as cartilage was clearly visible in the forelimb before birth but not in the hindlimb. By day 25 of pregnancy, the forelimb was well formed and the hindlimb shows early cartilage elements. Digits are numbered with Roman numerals.

At day 25, one day before birth, the forelimb had a well-formed elbow and keratinised epitrichial claws (Figure 2E). These claws were curved towards the ventral axis to form a fist and the living fetus repeatedly opened and closed the fist as well as moved the arms in a swimming motion. In contrast, the hindlimb was immobile and remained at right angles to the body. The hindlimb interdigital webbing had regressed and distinct digits were visible but the digits had not separated from each other (Table 2). However, the limbs had no ossification centres and contained only cartilaginous elements (Figure 3A and 3B) as detected using alcian blue and alizarin red staining, and by day 3 post-partum only the radius and ulna had begun to ossify (data not shown).

**Table 2:** Summary of limb development before birth in the tammar fetus

STAGE	FORELIMB	HINDLIMB
Day 18	Bud has formed with no defined shape as yet	Thickening of the hindlimb has occurred with a small protrusion as the beginnings of a bud
Day 19-21	Paddle like structure with the regions of the future digital rays beginning to form.	Elongation of the bud has occurred with no distinct structure
Day 22-23	Digital rays more pronounced and the interdigital tissue has begun to regress	The bud has formed into a flattened arrow-head like structure with the beginnings of the digital condensations. The pointed edge of the paddle appears to be the presumptive 4th digit
Day 23-24	Interdigital webbing has regressed. Epitrichial claws are present and fingers are open but not clenched. The distinct protrusion of the future elbow joint is beginning to form	The digital rays have begun to form and a distinct separation of the hindlimb digits begins. The interdigital webbing is reduced and an asymmetrical shape has started to form
Day 25	A well-defined forelimb with claws present with digits in a clenched position. The future elbow joint has become more pronounced	The interdigital webbing has regressed and all four digits are distinct. The 4th digit has become more pronounced and has an asymmetrical shape.

### Conservation and evolution of gene structure of *HOXA13 *and *HOXD13*

**Figure 4:**
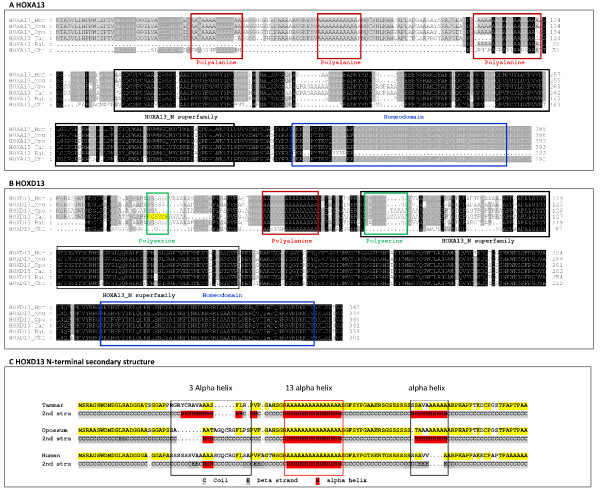
**(A and B) The conservation of the tammar HOXA13 (A) and HOXD13 (B) protein sequence**. Tammar protein is compared with human, mouse, opossum, bat and chicken orthologues. Shaded boxes show conservation of amino acids and crosses represent gaps in the sequence. Polyalanine tracts (red outline), polyserine tracts (green outline), homeodomains (blue outline) and HOXA13_N superfamily (black outline) are indicated by the boxed outlines. HOXD13 alignment of both tammar and opossum showed that the first polyserine repeats are missing in the tammar and the amino acids in this region differ markedly from the other 5 species shown (Figure 4B highlighted in yellow). Comparison of HOXD13 N-terminal secondary structure of this region (Figure 4C) showed that tammar produced a 13 α-helix structure (labelled with black frame) but the opossum and human only possess a 3 α-helix in the same region. Tammar, opossum and human all share a highly conserved α-helix structure (indicated by the red frame). Additionally another α-helix structure is present in tammar and opossum, but is absent in human (indicated by the black frame).

Both *HOXA13 *and *HOXD13 *have two exons that encoded 393 and 341 amino acids respectively. The HOX homeodomain was highly conserved in both *HOXA13 *and *HOXD13 *as in human, mouse, opossum, tammar, bat and chicken protein alignments (Figure 4A and 4B).

Tammar *HOXA13 *polyalanine tracts were highly conserved with those of human, bat and mouse, but not with the chicken. In contrast, the single tammar *HOXD13 *polyalanine tract was highly conserved in all species. However, the single opossum and tammar *HOXD13 *polyserine tract was conserved with the second of the two polyserine tracts that are present in human, mouse and bat (Figure 4B). Interestingly, there was a marked difference between the tammar and all the other mammals examined in the region of the first eutherian polyserine tract of *HOXD13*. It also differed markedly from this region in the opossum. Another region of difference was immediately downstream of the eutherian polyserine tract in which the amino acids GQCR are conserved in all species, including the opossum, but they are absent in the tammar (Figure 4B). In order to further compare the N-terminal region of HOXD13 in the human, opossum and tammar, the secondary structure was predicted and showed that the sequence in the tammar in the region of the first polyserine tract of the eutherians produced a 13-α-helix structure instead of the 3-α-helix structure that is present in both the human and opossum (Figure 4C). The conserved polyalanine tract formed a predicted α-helix structure but a third α-helix was also present in both opossum and tammar but absent from human (Figure 4C).

### *HOXA13 *and *HOXD13 *expression in the tammar autopod

**Figure 5:**
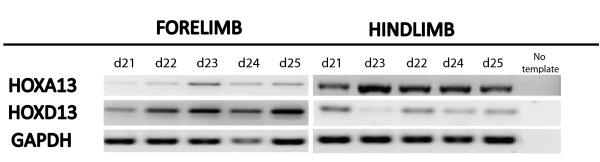
**A representative expression profile of *HOXA13 *and *HOXD13 *from day 21 to day 25 of pregnancy**. *HOXA13 *and *HOXD13 *was detected using RT-PCR at all stages in the fore and hindlimb stages (n = 3 for each stage).

**Figure 6:**
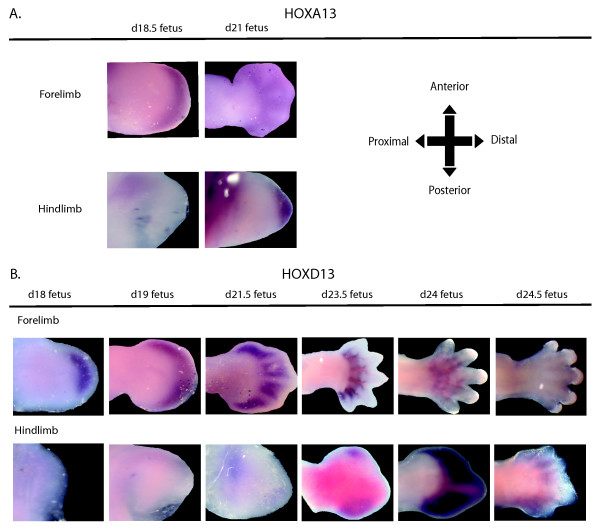
**mRNA wholemount *in situ *hybridisation expression of *HOXA13 *(A) and *HOXD13 *(B) in the tammar forelimb and hindlimb from day 18 to day 24.5 of pregnancy**. The top images for each gene are forelimbs with the corresponding hindlimb in the lower panel. Expression was first visible at day 18.5 in the forelimb. HOXA13 was expressed in the interdigital regions of the autopod by day 21 of pregnancy. Hindlimb mRNA was detected in the distal region of the hindlimb at day 21. *HOXD13 *in the forelimb was expressed distally from day 18 and became interdigital in the forelimb by day 21.5 of pregnancy. In the hindlimb, HOXD13 mRNA was not detected at day18 and 19, but was weakly expressed in the distal region at day 21.5. By day 23.5 was expressed in the anterior and posterior points of the limb and after day 24 to encompass the distal end of the hindlimb and became interdigital by day 24.5. All images are dorsal and orientated to point distally (n = 3).

RT-PCR was performed as an initial examination of *HOXA13 *and *HOXD13*. Both genes were detected from day 21 to day 25 of pregnancy in both the fore- and hindlimb (Figure 5). *HOXA13 *and *HOXD13 *expression during tammar limb development was also examined using wholemount *in situ *hybridisation (Figure 6). *HOXA13 *mRNA had an early and transient expression in the tammar autopod. Expression was first detected at day 18.5 in the distal margins of the forelimb paddle and extended from the anterior to the posterior margins. At the same stage there was no detectable expression in the hindlimb. Later at day 21, *HOXA13 *mRNA was restricted to the interdigital regions of the forelimb. The only detectable *HOXA13 *expression in the hindlimb was in the distal region of the hindlimb bud at day 21. In both the forelimb and hindlimb, *HOXA13 *transcripts was detected at day 24 and day 25.

*HOXD13 *expression was expressed distally in the day 18 and day 19 forelimb showing a similar expression pattern to the staining seen in *HOXA13 *at an equivalent stage (Figure 6B). There was no expression detected in the hindlimb at this stage. Two days later at day 21.5 *HOXD13 *was strongly expressed in the forelimb interdigital region and extended from the start of the digital condensations to the distal tip of the paddle. Unlike the forelimb, there was no detectable expression in the hindlimb bud.

At day 23.5, *HOXD13 *regressed towards the proximal boundary away from the tips of autopod. The first expression was detected in the hindlimb on this day of pregnancy and was expressed in the proximal and distal regions of the bud. At day 24, when the forelimb digits were clearly defined, the expression between the digits was weaker compared to day 23.5. Whilst expression was strongest in the hindlimb at this stage, *HOXD13 *was only expressed in the interdigital regions. By day 24.5, one day before birth, there was only weak hindlimb expression and no detectable expression in the forelimb. However, in these older and larger specimens this reduced expression may have been due to poor probe penetration.

### Tammar, mouse and chicken *HOXA13 *expression

**Figure 7:**
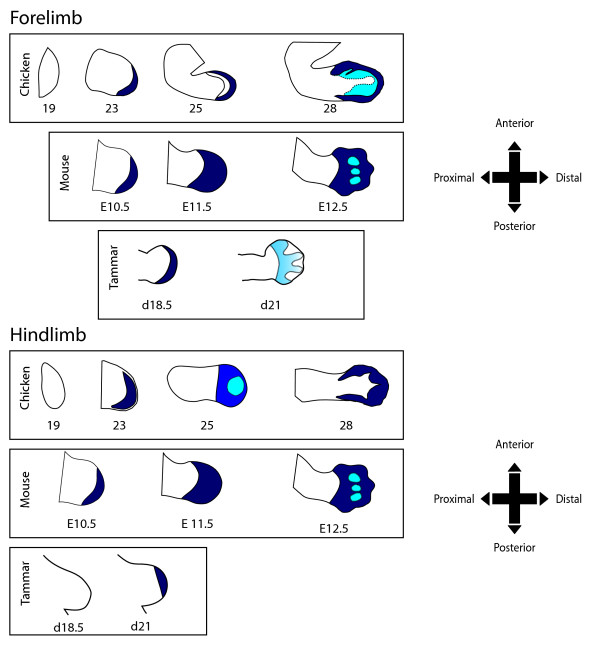
**Comparison of *HOXA13 *expression patterns in the mouse, chicken and tammar**. Patterns of mRNA expression in the mouse and chicken were traced from previously published data (Mouse: [43,54,55]; chicken:[42]). Strong staining is indicated in dark blue and weaker expression is represented in light blue. Dotted lines indicate digital rays.

The mouse and chicken *HOXA13 *expression pattern summarised diagrammatically from previous studies [40,41] were compared with that of the tammar (this study; Figure 7). *HOXA13 *was initially detected in day 19 of the tammar forelimb, whilst in the mouse expression is first detected at E10.5 [6] and at stage 23 in the chicken [42]. There is a similar distal expression pattern in the tammar, chicken and mouse forelimb and the expression has expanded proximally. At day 21, tammar *HOXA13 *was weakly expressed and restricted to the interdigital regions. The expression boundary had shifted the anterior-posterior boundary but this pattern was similar to that of both the mouse and chicken. At E11.5, mouse *HOXA13 *expands in the proximal direction and a day later at E12.5 the first signs of weaker expression are detected in the digital condensations [43]. This is similar to the chicken that that had expanded expression in the proximal direction by stage 25 [42]. At stage 28, the chicken wing has strong posterior margins and expression is excluded in the region where the elongated digit of the forelimb will form.

There was a restricted distal expression pattern of *HOXA13 *in the tammar hindlimb at day 21. In contrast, the chicken has a distal but expanded expression at stage 23 that was similar to the wing expression [42]. In contrast, the chicken leg at stage 25 has expanded expression towards the proximal axis and weakening expression in the distal region [42]. At stage 28 the chicken leg has strong and expanded distal expression around the anterior and posterior margins. There was no detectable expression where the digital condensations form. The mouse has similar fore- and hind- limb expression of *HOXA13 *[43] to the tammar and chicken forelimb.

### Tammar, mouse and chicken *HOXD13 *expression

**Figure 8:**
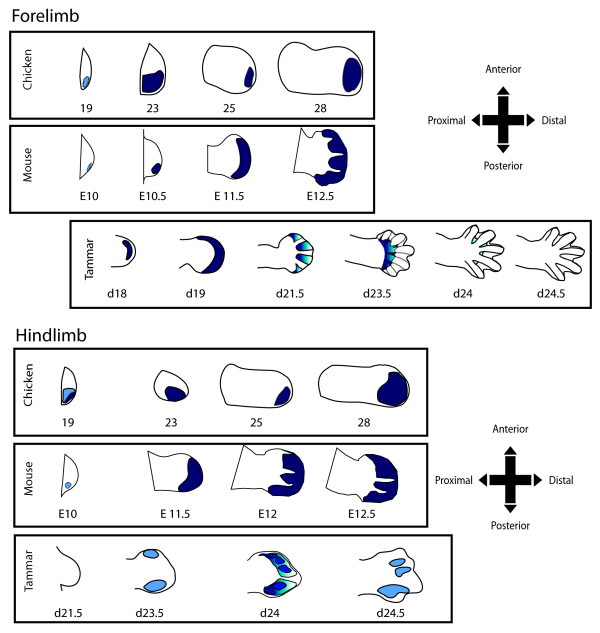
***HOXD13 *expression comparison between mouse, chicken and tammar**. Staining patterns were traced from published mRNA staining for mouse and chicken. (Mouse: [3,26]; chicken:[42]). Dotted lines indicate digital rays.

*HOXD13 *expression patterns between tammar, mouse and chicken were also compared (Figure 8). At day 18 in the tammar, *HOXD13 *expression was first observed in the forelimb in the stages examined and there was strong distal expression that extended from the anterior and posterior ends of the paddle like structure. This expression was similar to that of the mouse at E11.5 forelimb [3] although the first detectable expression in the mouse occurs at E10.0 in early bud stage and at stage 18 in the chicken [42]. At day 21.5, tammar *HOXD13 *became restricted to the interdigital regions, a pattern that was similar to the mouse E12.5 forelimb bud. *HOXD13 *in the chicken wing is more distally restricted and unlike expression in the tammar and mouse does not extend from the most anterior point to the posterior margins [42]. At day 23.5, *HOXD13 *in the tammar was less strongly expressed in the regions behind the digital condensations and in the interdigital regions. There is similar expression of *HOXD13 *in the chicken and mouse as in the staining of the corresponding tammar forelimbs. At day 24 *HOXD13 *was strongly expressed at the anterior and posterior margins with expression excluded where the digital condensations will form. A day later, one day before birth, there was no detectable expression.

## Discussion

Tammar *HOXA13 and HOXD13 *genes are highly conserved with those of the chicken and mouse but there were unique expression patterns of *HOXA13 *and *HOXD13 *in the developing limbs. In particular, the syndactylous digits of the hindlimb began their differentiation pre-natally, but there were marked differences in the time of expression of these genes in both the fore- and hindlimbs, supporting the suggestion that the HOX genes are as important for patterning of the marsupial autopod as they are in other mammals.

### The tammar limb shows heterochrony

The tammar forelimb is well advanced in the relative time of development compared to the mouse and chicken. At all stages, the development of the forelimb appears to be approximately two stages ahead of the hindlimb. This shift in developmental timing (heterochrony) is seen in other marsupial species like the opossum and the dasyurids [31]. However, the tammar has the most developed neonate amongst marsupials (Grade 3 as defined by Hughes and Hall, 1988) and has especially well-defined forelimbs that it uses to climb to the pouch. The control of heterochrony in marsupials remains unknown and an empirical analysis of tammar timing is not examined here, but in the opossum there is greater forelimb myocyte allocation compared with that of the mouse [29]. This heterochronic shift is a two-fold process with an acceleration of the forelimb and a delay in the development of the hindlimb bud [31]. However, post-natally, there is a rapid catch-up growth in the tammar hindlimb.

### The syndactylous digits are distinct in early development

The tammar hindlimb digits show clear evidence of syndactyly before birth. The rays of digits two and three are visible before any bone has formed in the hindlimbs. The early tammar fore and hindlimb buds both progress through a paddle-like stage, but the hindlimb soon becomes asymmetric. An early change in bud morphology is in accord with other species that also have reduced digits such as the chicken [42]. The tammar precursors of pedal digit 1 are not present at any stage, and the tammar hindlimb bud is more asymmetric than the chicken that has lost 2 digits. A reduction in the allocation of cells destined for the condensation of the missing digit may explain its loss [44]. Across different dog breeds there is variability in digit number and the number of digits may be due to the variability of the size of the breed and the number of cells in the embryonic limb [44].

### Marsupial *HOXA13 *and *HOXD13 *are highly conserved

Tammar hindlimbs are reminiscent of mice and humans that have *HOXA13 *and *HOXD13 *mutations. However, the marsupial gene structure is highly conserved with mouse and opossum except for the N-terminal region of *HOXD13*. As expected, tammar *HOXA13 *and *HOXD13 *are highly conserved within all major vertebrate groups analysed, a phylogenetic tree (see Additional file 2) constructed with the predicted amino acids and others retrieved from NCBI (http://www.ncbi.nlm.nih.gov/protein) or Ensembl (http://asia.ensembl.org/index.html) produced tight branches. Given the high conservation of gene structure the specialised marsupial hindlimb is likely to be influenced through modification of gene expression domains, and timing or changes to downstream targets.

In mice lacking HOXA13, there are two potential downstream targets, *Gdf5 *and *Bmp2 *[45]. *Gdf5 *and *Bmp2 *are up- and down- regulated respectively and have diffuse expression in the carpal and tarsal anlagen. Interestingly, these mice also have a range of phenotypes including a fusion of carpal or tarsal elements and an absence of digit 1 in the hindlimb [16,45], a phenotype somewhat similar to that of the tammar hindlimb. *HOXA13 *is also expressed at an equal level in all digits of the limb including digit 1. Macropodid marsupial hindlimbs lack digit 1 and have fused tarsals, so by analogy HOXA13 is likely to have been involved in the evolution of these kangaroo phenotypes.

Determining the regulatory control of HOX genes is crucial in understanding the evolution of the different digital forms. The regulation of the HOX cluster is believed to be controlled by a quantitative mechanism involving both gene topography and dosage. The relative proximity of *HOX *genes to two enhancers PROX and GCR located on the 5' end determines how strongly *HOX *genes are expressed [46]. In mice *HOXD13 *is located towards the 5' end of the cluster and is expressed lower in the region destined to become digit one. In contrast, in the tammar forelimb which has a digit 1, we did not detect a difference in expression of *HOXD13 *in any digit. Interestingly, in the hindlimb with its developing syndacytlous digits, there is a lack of expression in the region of the prospective digit 4. This is similar to the situation observed in the bat and chick where 5'HOXD expression is restricted to the interdigital regions and excluded from condensing mesenchyme [26,47] In addition, HOXD expression is believed to play a role in determining digit size and number through a dose dependant mechanism [48].

Interestingly, there has been at least one report of polydactyly in the forelimb of a kangaroo [49], a phenotype reminiscent of mutations in human *HOXA13 *and *HOXD13*. Expansions or deletion in the polyalanine tracts of *HOXA13 *and *HOXD13 *is associated with these mutant phenotypes in man and mouse [50]. However, in dogs the variation in the number of tandem repeats in the genome is correlated with changes in limb and skull form [51]. In particular, the change in repeat length in Aristaless-like 4 (ALX4) observed between different dog species, was associated with formation of a rear first polydactyl digit [51].

Other vertebrate groups such as cetaceans have a novel expansion of the polyalanine tract in *HOXD13 *compared with humans and mice, indicating it may influence the morphological diversity of the cetacean autopod [52]. Chicken and *Zebrafish *polyalanine tracts are shorter and less frequent compared to those of mammals. However, most polyalanine mutations lead to protein mis-folding, degradation and cytoplasmic aggregation and can repress expression depending on the number of polyalanines [19,50]. Bats have highly modified forelimbs with a greatly elongated third digit and have shifted the *HOXD13 *anterior-posterior limits in the forelimb compared to the mouse but retain conserved polyalanine tracts [50]. The tammar has also shifted the HOXD13 anterior-posterior boundaries (in the hindlimb), but does not have any mutation in the first and third polyalanine tracts (Figure 4) and so this cannot account for the unique tammar hindlimb. However, the first polyserine tract was missing in the N-terminal of HOXD13 in both tammar and opossum, and instead possessed a unique series of amino acids in the tammar. It is possible that these six amino acids could affect the limb phenotype. The secondary structure of HOXD13 in the tammar included a long 13 α-helix, but in the human and the opossum it was only a 3 α-helix (Figure 4), suggesting that this region may be important in the development of a macropodid-specific form.

## Conclusions

This study is the first to describe *HOX *expression in any marsupial. The subtle differences in gene structure in the tammar and the changes in expression and timing may drive the differences in the development of the syndactylous limb. *HOXA13 *and *HOXD13 *gene structures are highly conserved between marsupial, chicken and mouse. The lack of polyalanine modifications suggests these regions in either gene are unlikely to be the cause of altered limb morphology in the tammar but that the polyserine region may well be responsible for the development of marsupial syndactyly. Our findings support the hypothesis that changes to the structure and function of *HOXA13 *and *HOXD13 *affect regulation of digit identity in this marsupial.

## Authors' contributions

The authors declare the following contributions: conceived the study and design of experiments: KYC, HY and MBR. Collected tissues: KYC, HY, GS and MBR. Performed the experiments and analysis: KYC, HY and MBR. Manuscript preparation and discussion: KYC, HY, AJP and MBR. All authors read and approved the final manuscript.

## Supplementary Material

Additional file 1 **Table S1: Protein sequences of HOXA13 and HOXD13**. The sequences used in this study were retrieved from GenBank or Ensembl. Click here for file

Additional file 2 **Phylogenetic tree of HOXA13 and HOXD13**. The evolutionary history was inferred using the Neighbor-Joining method [56]. The bootstrap consensus tree inferred from 1000 replicates is taken to represent the evolutionary history of the taxa analyzed [57]. Branches corresponding to partitions reproduced in less than 50% bootstrap replicates were collapsed. The percentage of replicate trees in which the associated taxa clustered together in the bootstrap test (1000 replicates) are shown next to the branches [57]. The evolutionary distances were computed using the Poisson correction method [37] and are in the units of the number of amino acid substitutions per site. The analysis involved 24 amino acid sequences with a total of 114 positions in the final dataset. All positions containing gaps and missing data were eliminated. Evolutionary analyses were conducted in MEGA5 [37]. Click here for file
