# Supplementary material for: HOXA13 and HOXD13 expression during development of the syndactylous digits in the marsupial Macropus eugenii
Source: BMC Dev Biol. 2012 Jan 11;12:2. doi: 10.1186/1471-213X-12-2 (PMC3268106; doi:10.1186/1471-213X-12-2)
Supplement: Additional file 1 — Table S1: Protein sequences of HOXA13 and HOXD13. The sequences used in this study were retrieved from GenBank or Ensembl. [file 1471-213X-12-2-S1.PDF]

Species	HOXA13	*HOXD13*
Human	NP_000513	NP_000514
mouse	NP_032290.1	NP_032301
Rat	XP_575481	NP_001099356
Rabbit	ENSOCUP00000019213	XP_002712251
Bat	ENSMLUP00000007601	AAW66480
Dog	ENSCAFP00000004403	-
Kangaroo Rat	ENSDORP00000005921	-
Opossum	XP_001362476	Predicated
Platypus	XP_001510976	ENSOANP00000007506
Chicken	NP_989470	NP_990765
Frog	NP_001079321	NP_001083950
Zebrafish	ENSDARP00000052661	CAA61031
