# Supplementary figures and images for: HOXA13 and HOXD13 expression during development of the syndactylous digits in the marsupial Macropus eugenii
Source: BMC Dev Biol. 2012 Jan 11;12:2. doi: 10.1186/1471-213X-12-2 (PMC3268106; doi:10.1186/1471-213X-12-2)

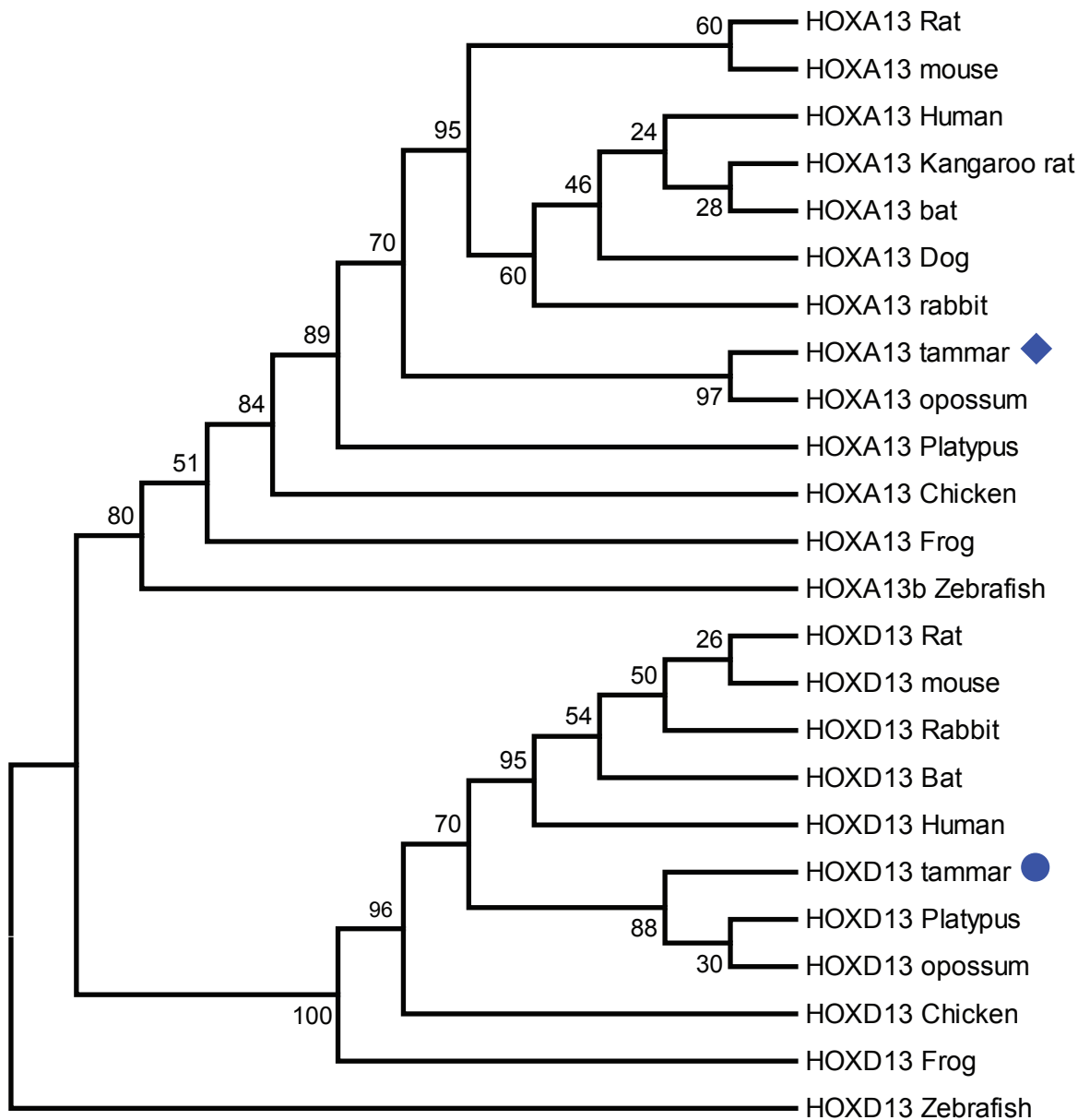

Supplement: Additional file 2 — Phylogenetic tree of HOXA13 and HOXD13. The evolutionary history was inferred using the Neighbor-Joining method [56]. The bootstrap consensus tree inferred from 1000 replicates is taken to represent the evolutionary history of the taxa analyzed [57]. Branches corresponding to partitions reproduced in less than 50% bootstrap replicates were collapsed. The percentage of replicate trees in which the associated taxa clustered together in the bootstrap test (1000 replicates) are shown next to the branches [57]. The evolutionary distances were computed using the Poisson correction method [37] and are in the units of the number of amino acid substitutions per site. The analysis involved 24 amino acid sequences with a total of 114 positions in the final dataset. All positions containing gaps and missing data were eliminated. Evolutionary analyses were conducted in MEGA5 [37]. [file 1471-213X-12-2-S2.PDF]
